# Low n-3/High n-6 Flaxseed and *Hermetia illucens* Larvae Oils as Feed Materials: Effects on In Vitro Rumen Fermentation, Maternal Metabolism, Antioxidant Status and Colostrum in Prolific Ewes

**DOI:** 10.3390/ijms27188111

**Published:** 2026-09-11

**Authors:** Kamila Lewandowska, Natalia Pachura-Hanusek, Anna Burek, Antoni Szumny, Robert Kupczyński

**Affiliations:** 1 Department of Environmental Hygiene and Animal Welfare, Faculty of Biology and Animal Science, Wrocław University of Environmental and Life Sciences, Chełmońskiego St. 38c, 51-630 Wrocław, Poland; natalia.pachura@upwr.edu.pl (N.P.-H.); anna.burek@upwr.edu.pl (A.B.); robert.kupczynski@upwr.edu.pl (R.K.); 2 Department of Food Chemistry and Biocatalysis, Faculty of Biotechnology and Food Science, Wrocław University of Environmental and Life Sciences, Norwida St. 27, 50-375 Wrocław, Poland; antoni.szumny@upwr.edu.pl

**Keywords:** black soldier fly larvae oil, flaxseed oil, sheep colostrum, rumen fermentation, fatty acids, methane concentration, antioxidant status, transition period, prolific ewes

## Abstract

Black soldier fly larvae (BSFL; *Hermetia illucens*) oil and flaxseed oil provide contrasting fatty acid profiles and may serve as alternative lipid feed materials in ruminant nutrition. This study assessed low-α-linolenic/high-linoleic flaxseed oil, BSFL oil, and their blends using an *in vitro* rumen fermentation screening with pooled bovine rumen inoculum and a randomized prepartum feeding trial in 32 twin- or triplet-bearing Olkuska ewes. *In vitro*, the single oils and a 1:1 blend were evaluated at two inclusion levels; total VFA concentration was unchanged, whereas propionate proportion, the acetate-to-propionate ratio, methane, ammonia, and incubation-fluid fatty acid profiles showed treatment-dependent responses. *In vivo*, an unsupplemented control was compared with flaxseed oil, BSFL oil, and a 2:1 blend supplied at 30 g/day for 21 days before expected parturition. Colostrum fat concentration differed among treatments (*p* = 0.036); the 2:1 blend group had a higher value than the control group (11.29% vs. 8.26%; Dunnett-adjusted *p* < 0.05), while major fatty acid classes did not differ. Supplementation was also associated with differences in selected minor colostrum fatty acids and several serum metabolic variables. Colostrum yield was not measured; therefore, this finding reflects fat concentration rather than total colostrum fat output. Serum total antioxidant status (TAS) was unaffected, while a treatment-by-time interaction was observed for γ-glutamyl transferase. The tested plant- and insect-derived oils therefore modified ruminal lipid transformations and selected maternal and colostrum traits without a detectable change in TAS.

## 1. Introduction

The transition from late gestation to early lactation imposes substantial metabolic demands on prolific ewes. Rapid fetal growth, reduced rumen capacity, and the onset of colostrum synthesis may contribute to a negative energy balance, mobilization of body reserves, and the development of pregnancy toxemia, particularly in ewes carrying multiple fetuses [1,2,3,4]. The Olkuska ewe is a prolific Polish breed characterized by frequent multiple pregnancies [5]. These metabolic adaptations are closely associated with hepatic lipid metabolism and redox homeostasis. Therefore, dietary strategies for transition ewes should be assessed not only in terms of their effects on energy-related blood metabolites and colostrum composition, but also their potential influence on systemic antioxidant status.

Dietary lipids are functional feed ingredients whose fatty acid composition may affect rumen microorganisms, biohydrogenation pathways, fermentation products, and the fatty acids available for intestinal absorption [6,7,8,9,10,11,12,13,14,15]. Polyunsaturated fatty acids may modify microbial membrane properties and rumen fermentation, whereas medium-chain fatty acids may exert selective antimicrobial effects. In transition-ewe nutrition, lipid supplementation is also used to modify the fatty acid composition of colostrum and milk [16,17,18,19]. However, these responses depend strongly on the lipid source and dose. Thus, a lipid supplement that alters ruminal fatty acid transformations may not necessarily improve energy metabolism or antioxidant status *in vivo*.

Black soldier fly larvae (BSFL; *Hermetia illucens*) oil is an emerging insect-derived feed ingredient characterized by a high proportion of lauric acid [20,21,22,23]. Flaxseed oil is generally characterized by a high proportion of α-linolenic acid [24,25]; however, its fatty acid composition is strongly influenced by flax genotype, and low-α-linolenic/high-linoleic types are also available. The flaxseed oil evaluated in the present study represented such a fatty acid profile. Because linoleic and α-linolenic acids differ in their ruminal biohydrogenation kinetics [26], this flaxseed oil and the lauric-acid-rich BSFL oil represented compositionally contrasting substrates for rumen lipid metabolism.

Ewe colostrum composition is particularly important for lambs from multiple births. Colostral fat provides energy required for thermoregulation and early postnatal adaptation, while other components support passive immunity and growth [27,28,29]. Interest in BSF-derived feed ingredients for ruminants has increased substantially in recent years, although *in vivo* evidence remains limited, particularly for BSFL oil and sheep [21,23,30,31]. Recent studies indicate that responses may vary depending on the form of the BSF-derived product, inclusion level, animal species, and experimental conditions [31,32]. In lactating dairy ewes, replacement of palm-derived fat with BSFL oil maintained animal performance and most *in vivo* ruminal fermentation parameters, but was associated with higher *in vitro* methane production and lower serum antioxidant capacity [33]. In young ruminants, BSFL oil has also been shown to partially replace conventional lipid sources without adversely affecting growth or feed efficiency [34]. However, direct comparisons of BSFL oil and flaxseed oil with respect to rumen fermentation responses in sheep remain scarce. Information is also limited on the transfer of dietary fatty acids into colostrum following lipid supplementation during late gestation in ewes. Moreover, the combined supplementation of BSFL oil and flaxseed oil with a low-α-linolenic/high-linoleic fatty acid profile has not yet been evaluated in prolific ewes during the transition period.

The present study therefore comprised two complementary experiments. First, flaxseed oil, BSFL oil, and their 1:1 blend were screened *in vitro* to determine their effects on rumen fermentation and fatty acid profiles measured by gas chromatography–mass spectrometry (GC–MS). Second, flaxseed oil, BSFL oil, and their 2:1 blend were evaluated in a randomized prepartum feeding trial. The trial assessed their effects on maternal serum metabolism, liver-related biochemical variables, total antioxidant status, and colostrum composition. We hypothesized that these contrasting lipid sources would induce source-dependent changes in ruminal lipid transformations and selected maternal and colostrum characteristics without substantially reducing the production of the main rumen fermentation products.

## 2. Results

### 2.1. In Vitro Rumen Fermentation

#### 2.1.1. Fatty Acid Composition of the Experimental Oils

The fatty acid compositions of the specific oil batches used in this study were determined by gas chromatography using the procedure described in Section 4.5.2.

Unlike conventional flaxseed oil, the flaxseed oil used in this study exhibited an unusual fatty acid composition characterized by a high proportion of n-6 polyunsaturated fatty acids (PUFA). Linoleic acid (C18:2 n-6 cis) was the predominant fatty acid, accounting for 54.57% of total fatty acids, whereas α-linolenic acid (C18:3 n-3) represented only 2.95%. Other major fatty acids included palmitic acid (C16:0; 16.20%), oleic acid (C18:1 n-9 cis; 13.61%), and stearic acid (C18:0; 11.42%).

In contrast, BSFL oil was dominated by saturated fatty acids, particularly medium-chain fatty acids. A high proportion of lauric acid is characteristic of BSFL oil, although its fatty acid composition may vary with larval diet and production conditions [33,34]. Lauric acid (C12:0) was the most abundant fatty acid, accounting for 51.71% of total fatty acids. Palmitic acid (C16:0) and myristic acid (C14:0) constituted 19.52% and 11.73%, respectively. Unsaturated fatty acids were present at lower proportions, with linoleic acid (C18:2 n-6 cis) and oleic acid (C18:1 n-9 cis) accounting for 5.36% and 5.26%, respectively. The GC–MS profiles therefore confirmed that the experimental oils represented compositionally distinct plant- and insect-derived lipid feed materials.

#### 2.1.2. Fermentation Characteristics and Methane

At 4 h, pH differed among treatments (*p* = 0.001), while differences at 24 h were smaller (*p* = 0.022). NH_3_-N concentration differed among treatments at both incubation times (*p* < 0.001).

Total VFA concentration and the proportions of acetate and butyrate were unaffected by treatment. Propionate proportion and the acetate-to-propionate ratio differed among treatments after 24 h (*p* < 0.001 and *p* = 0.001, respectively), with lower acetate-to-propionate ratios in the 800 mg BSFL oil and 800 mg blend treatments.

Methane concentration differed among treatments at both 4 and 24 h (*p* < 0.001), showing source- and dose-dependent responses (Table 1).

#### 2.1.3. *In Vitro* Fatty Acid Profiles

After 4 h, the fatty acid profile of the incubation fluid differed among treatments (Table 2). The high lauric acid content of BSFL oil was reflected in the BSFL and blend treatments. Flaxseed-oil treatments showed higher proportions of linoleic acid, α-linolenic acid, vaccenic acid, and total unsaturated fatty acids at selected doses. Because the fatty acid values are expressed as proportions of total identified fatty acids, increases in one component necessarily affect the relative proportions of others.

Stearic acid was lower with 800 mg BSFL oil than in the control, while the remaining treatments showed intermediate values. Odd- and branched-chain fatty acid proportions were generally lower in treatments containing BSFL oil or the blend. These findings describe treatment-specific changes in the incubation fluid and should not be interpreted directly as the fatty acid composition of animal products.

The n-6/n-3 ratio varied markedly among treatments, reflecting the contrasting composition of the two oils. The highest ratio at 4 h occurred with 800 mg BSFL oil, while the 1600 mg blend had an intermediate value.

After 24 h, treatment differences in the fatty acid profile remained evident (Table 3). Lauric acid remained high in the BSFL-oil treatments, and vaccenic acid was highest with 400 mg flaxseed oil. α-Linolenic acid was lower with 800 mg BSFL oil than with 400 mg flaxseed oil, while the remaining treatments were intermediate.

The n-6/n-3 ratio was highest with 800 mg BSFL oil; the control showed a lower value, while the remaining treatments were intermediate. These results reflect the different fatty acid inputs and their transformations during incubation; they do not establish a nutritional advantage of a particular treatment *in vivo*.

### 2.2. In Vivo Feeding Trial

The *in vivo* findings are presented independently of the *in vitro* screening because the blend ratio differed between experiments (1:1 *in vitro* and 2:1 *in vivo*).

#### 2.2.1. Colostrum Composition

Dietary treatment affected colostrum fat concentration (*p* = 0.036; Table 4). The 2:1 blend group had a higher fat concentration than the control (11.29% vs. 8.26%). Solids-not-fat, density, total protein, lactose and mineral salts did not differ among groups.

#### 2.2.2. Colostrum Fatty Acid Profile

The effect of prepartum maternal supplementation with different fat sources on the fatty acid profile of colostrum is presented in Table 5. Dietary treatment did not significantly affect the proportions of the major fatty acid classes or the calculated lipid quality indices. Significant overall treatment effects were observed only for selected individual fatty acids, namely C13:0, iso-C16:0, anteiso-C16:0, α-linolenic acid (C18:3 n-3; ALA), and arachidonic acid (C20:4 n-6; ARA), as well as for the total proportion of odd- and branched-chain fatty acids (OBCFA).

Prepartum supplementation with flaxseed oil, BSFL oil, or their mixture did not significantly affect the proportions of medium-chain fatty acids (MCFA; *p* = 0.486), long-chain fatty acids (LCFA; *p* = 0.483), saturated fatty acids (SFA; *p* = 0.741), monounsaturated fatty acids (MUFA; *p* = 0.763), polyunsaturated fatty acids (PUFA; *p* = 0.267), or total unsaturated fatty acids (UFA; *p* = 0.745). Likewise, no treatment effects were detected for n-6 PUFA, n-3 PUFA, the n-6/n-3 or n-3/n-6 ratios, or the atherogenicity and thrombogenicity indices (*p* > 0.05). The proportions of odd-chain fatty acids (OCFA; *p* = 0.059) and branched-chain fatty acids (BCFA; *p* = 0.052) were also not significantly affected by dietary treatment.

Significant differences were found for selected individual fatty acids. The proportion of anteiso-C16:0 was lower in all oil-supplemented groups than in the control group (*p* = 0.002). The proportion of iso-C16:0 was lower in the BSFL group than in the control group, whereas the flaxseed oil and blend groups had intermediate values and did not differ significantly from either group (*p* = 0.026). ARA was lower in the BSFL and blend groups than in the control group, while the flaxseed oil group showed an intermediate value (*p* = 0.014).

An overall treatment effect was also detected for C13:0 (*p* = 0.043) and ALA (*p* = 0.048). However, no significant pairwise differences among treatments were identified for these fatty acids using the Tukey–Kramer test. The total OBCFA proportion was lower in the flaxseed oil and BSFL groups than in the control group, whereas the blend group had an intermediate value (*p* = 0.019). Overall, prepartum oil supplementation modified the proportions of several individual and minor fatty acids but did not alter the general distribution of the major fatty acid classes or the lipid quality indices of colostrum.

### 2.3. Maternal Metabolic and Antioxidant Responses

The baseline-adjusted analysis (Table 6) showed that the treatment and time of sample collection had an effect on serum GLU concentration (*p* = 0.003 and *p* = 0.008, respectively), while no treatment × time interaction was observed (*p* = 0.766). Across the post-baseline period, GLU was lower in the Control group than in the Flaxseed oil group, and concentrations were higher at 21 days postpartum than at 7 days postpartum. NEFA showed a modest time effect (*p* = 0.047), but neither the treatment effect nor the interaction was significant (*p* = 0.262 and *p* = 0.405); no pairwise time comparisons remained significant after Holm adjustment. BHB was affected by treatment and time (*p* = 0.042 and *p* = 0.001), without an interaction (*p* = 0.191). Although the overall treatment effect was significant, no marginal treatment pairwise comparison remained significant after adjustment; BHB was lower at the end of supplementation than at both postpartum sampling times.

TG showed a treatment × time interaction (*p* = 0.014) and a pronounced time effect (*p* < 0.001), whereas the overall treatment effect was not significant (*p* = 0.128). At the end of supplementation, TG was higher in the blend group than in the control group, followed by a decline after parturition. TC was affected by treatment and time (*p* = 0.012 and *p* < 0.001), without an interaction (*p* = 0.536); the blend group had higher marginal TC than the other treatments, and values were highest at the end of supplementation. HDL-C also showed treatment and time effects (*p* = 0.045 and *p* < 0.001), with no interaction (*p* = 0.430); marginal HDL-C was higher in the blend group than in the flaxseed oil group and was lowest at 7 days postpartum. LDL-C was affected only by time (*p* < 0.001), decreasing progressively after the end of supplementation, with no treatment or interaction effects (*p* = 0.362 and *p* = 0.670).

AST activity was affected by sampling time (*p* < 0.001), but not by treatment or the treatment × time interaction (*p* = 0.432 and *p* = 0.569, respectively; Table 7). Baseline-adjusted AST activity increased from the end of supplementation to 7 and 21 days postpartum. ALT also varied with time (*p* = 0.030), with higher activity at the end of supplementation than at 7 days postpartum, whereas treatment and interaction effects were not significant (*p* = 0.972 and *p* = 0.598). GGT showed a treatment × time interaction (*p* = 0.006) and a time effect (*p* < 0.001), although the overall treatment effect was not significant (*p* = 0.177). At 21 days postpartum, GGT activity was higher in the blend group than in the control and BSFL-oil groups; the blend did not differ from flaxseed oil. ALP showed a numerical tendency to vary with time (*p* = 0.069), and total bilirubin was affected only by time (*p* < 0.001). Total antioxidant status was not affected by treatment, time, or their interaction (*p* = 0.691, *p* = 0.192, and *p* = 0.567, respectively), providing no evidence of a supplementation-induced change in this global marker of systemic antioxidant capacity.

Table 8 shows that neither treatment, time, nor the treatment × time interaction had a significant effect on TP (*p* = 0.069, *p* = 0.251, and *p* = 0.814, respectively), although a numerical tendency for a treatment effect was observed. ALB was affected only by time (*p* < 0.001), with lower concentrations at 21 days postpartum than at the end of supplementation and 7 days postpartum; treatment and interaction effects were not significant (*p* = 0.808 and *p* = 0.327). BUN also varied with time (*p* = 0.014), being lower at 7 days postpartum than at the end of supplementation, whereas treatment and interaction effects were not significant (*p* = 0.838 and *p* = 0.617).

CREA was affected by treatment, time, and their interaction (*p* = 0.012, *p* < 0.001, and *p* = 0.030, respectively). At the end of supplementation, CREA was higher in the BSFL oil and Blend groups than in the Control and Flaxseed oil groups. CREA subsequently declined in the Control, BSFL oil, and Blend groups, and no treatment differences were detected at either postpartum sampling time.

## 3. Discussion

This study evaluated compositionally contrasting plant- and insect-derived oils as alternative lipid feed materials using *in vitro* rumen screening and a randomized prepartum feeding trial in prolific ewes. The principal *in vivo* finding was a higher colostrum fat concentration in ewes receiving the 2:1 flaxseed-to-BSFL oil blend. The major colostrum fatty acid classes were unchanged, maternal biochemical responses were selective and time-dependent, and total antioxidant status did not respond to supplementation.

The absence of significant treatment effects on total VFA concentration and the proportions of acetate and butyrate indicates that the tested lipid additions did not markedly suppress overall fermentation. However, propionate proportion and the acetate-to-propionate ratio differed among treatments after 24 h, indicating a shift in fermentation pattern rather than an improvement in fermentation efficiency. Recent *in vitro* evidence similarly indicates that flaxseed-derived lipid supplementation can modify gas and methane production without markedly altering total VFA concentration or the major VFA proportions [35]. Nevertheless, changes in ammonia, the acetate-to-propionate ratio, methane, and the GC–MS-derived fatty acid profiles show that the treatments were biologically active. Dietary lipids can alter ruminal microbial activity and biohydrogenation, with responses determined by fatty acid structure and inclusion level [6,7,8,9,10,11,12,13,14,15]. Recent meta-analytic evidence indicates that BSF-derived feed ingredients may modify rumen fermentation without consistently affecting individual VFA proportions or ruminal pH, although an increase in total VFA concentration has been reported [31]. *In vivo* evidence in sheep also indicates that linseed oil supplementation can modify ruminal fermentation and microbial community structure, supporting the view that responses to plant-derived lipids depend on fatty acid composition and dietary conditions [36]. The lower acetate-to-propionate ratio observed for selected treatments should therefore be interpreted as a shift in fermentation pattern rather than direct evidence of improved energetic efficiency.

The incubation-fluid fatty acid profiles reflected both the original composition of the oils and their subsequent ruminal transformations. BSFL-oil treatments retained high proportions of lauric acid, whereas flaxseed-oil treatments increased linoleic acid, α-linolenic acid, vaccenic acid, and total unsaturated fatty acids at selected doses and time points. The reduction in stearic acid and in several odd- and branched-chain fatty acids in treatments containing BSFL oil is consistent with a substantial alteration of the relative fatty acid pool. Because the data are compositional and were obtained using pooled bovine inoculum, they provide mechanistic screening information but cannot be translated directly into predictions of colostrum fatty acid composition in ewes. Previous meta-analytic evidence indicates that the effects of dietary fat on digestibility and several rumen fermentation responses are broadly comparable between cattle and sheep, although the magnitude of the methane response may differ between species [15]. Therefore, the bovine inoculum was considered suitable for qualitative screening, while species-specific extrapolation to sheep remains limited. Furthermore, the *in vitro* substrate (hay and wheat grain, 1:1) differed from the basal diet used *in vivo*, which consisted of meadow hay and a compound concentrate, and the lipid inclusion levels applied *in vitro* cannot be directly converted to the 30 g/day supplementation used in ewes. Accordingly, the in vitro experiment should be regarded as a qualitative screening approach for identifying source- and dose-dependent fermentation responses rather than as a quantitative predictive model of the subsequent in vivo response.

Methane responses were non-linear and strongly dose-dependent. Recent *in vivo* evidence in dairy ewes likewise indicates that responses to BSFL oil may depend on experimental conditions, as Baila et al. reported unchanged in vivo ruminal fermentation parameters but higher methane production in an *in vitro* assay using inoculum from BSFL-oil-fed ewes [33]. The response of ruminal methane production to flaxseed-derived supplements appears to depend on the form of the lipid source, inclusion level, and basal diet composition, as also indicated by recent *in vitro* work [35]. Both flaxseed-oil doses and selected BSFL-oil and blend treatments reduced methane relative to the control, whereas 800 mg BSFL oil and 800 mg blend increased it. This pattern does not support a general antimethanogenic claim for BSFL oil. Instead, it indicates that the interaction between lauric-acid-rich insect oil, unsaturated plant oil, and total lipid dose can redirect microbial fermentation in different directions [8,9,10,11,12,14,15,37]. The mechanism underlying this non-linear response remains unclear and may reflect dose-dependent shifts in rumen microbial activity and fermentation pathways.

The 2:1 oil blend increased colostrum fat concentration by approximately 37% relative to the control. Interestingly, a recent meta-analysis of BSF-derived ingredients in ruminants reported an increase in milk fat percentage, whereas milk yield was not significantly affected [31]. Higher colostrum energy density could be relevant to multiple-birth lambs, which have limited energy reserves and are susceptible to hypothermia and starvation [27,28,29]. However, the present result concerns fat concentration rather than total colostrum fat yield. Colostrum yield, immunoglobulin concentration, passive transfer, and neonatal outcomes were not measured, and the diets were not energy-matched. Therefore, it cannot be determined whether the higher fat concentration reflected differences in total fat secretion or in colostrum volume, and the present data do not allow the direction of any potential effect of additional dietary energy on colostrum yield to be established. Consequently, the finding should be interpreted as a modification of the energy-related composition of colostrum, not as evidence of improved overall colostrum quality or lamb survival.

The proportions of the major saturated, monounsaturated, and polyunsaturated fatty acid classes in colostrum were not altered. Responses were confined mainly to individual minor fatty acids, including arachidonic acid, and to odd- and branched-chain fatty acids. Given their low relative abundance and the absence of changes in the major fatty acid classes, the nutritional relevance of these differences for neonatal lambs remains to be established. The limited correspondence between the pronounced *in vitro* fatty acid changes and the colostrum profile is consistent with extensive ruminal modification of dietary unsaturated fatty acids through biohydrogenation. In the present *in vitro* system, changes in linoleic and α-linolenic acids, together with the accumulation of vaccenic acid (C18:1 trans-11), an intermediate of ruminal biohydrogenation, indicate active transformation of dietary PUFA. However, because fatty acids were expressed as relative proportions rather than absolute amounts, the apparent extent of biohydrogenation could not be quantified reliably. Following ruminal metabolism, the relationship between dietary fatty acids and colostrum composition may be further attenuated by their incorporation into circulating and endogenous lipid pools, mobilization of body fat during the periparturient period, and mammary de novo fatty acid synthesis [26,31,32,33,34,35,36]. Similar variability in milk fatty acid responses to lipid supplementation has been reported across ruminant species and oil sources [38,39,40,41].

Serum glucose, β-hydroxybutyrate, total cholesterol, HDL-C, triglycerides, and creatinine showed treatment- or treatment-by-time responses, whereas NEFA and several liver, protein, and renal indicators were driven mainly by the physiological transition from late gestation to lactation. These results indicate modulation of selected aspects of circulating lipid and energy metabolism, but they do not demonstrate a consistent reduction in adipose-tissue mobilization or the risk of pregnancy toxemia. The lack of individual feed-intake measurements and the absence of an energy-matched control prevent the separation of fatty-acid-specific effects from the effect of additional dietary energy.

Serum TAS was the only antioxidant endpoint assessed in the present study, and no significant differences among dietary treatments were observed. Individual enzymatic and non-enzymatic components of the antioxidant defense system were not measured. Therefore, the absence of a treatment effect on serum TAS should be interpreted specifically within the scope of this integrated serum measure and does not exclude potential effects on individual components of antioxidant defense. Evidence from lactating ewes also does not indicate a consistent antioxidant response to BSFL oil supplementation. Baila et al. reported lower serum antioxidant capacity in BSFL-oil-fed ewes, whereas reactive oxygen metabolite concentrations remained unchanged [33]. Thus, the current data do not support either an antioxidant or a pro-oxidant effect of the supplements on systemic antioxidant status as measured by this assay. The isolated increase in GGT activity in the blend group at 21 days postpartum was not accompanied by treatment effects on AST, ALT, bilirubin, or total antioxidant status. It should therefore be interpreted as a selective biochemical response rather than evidence of generalized hepatic injury or altered redox homeostasis.

The main strengths of the study are the use of GC–MS to characterize ruminal and colostral fatty acid responses, the randomized *in vivo* design, repeated baseline-adjusted serum measurements, and the inclusion of a prolific sheep breed with high metabolic demands. The colostrum results describe compositional changes rather than total colostrum output; the dietary treatments were not energy-matched; and total antioxidant status was used as an integrative marker of circulating antioxidant capacity without additional redox-related markers. In addition, the *in vitro* phase was intended as a qualitative screening approach using pooled bovine inoculum and should not be interpreted as a direct quantitative model of the subsequent *in vivo* response. Within this framework, the study provides an integrated assessment of how two compositionally distinct alternative lipid feed materials influence ruminal lipid transformations, maternal biochemistry, and colostrum composition.

## 4. Materials and Methods

### 4.1. Study Design

The study comprised an *in vitro* screening experiment and a randomized feeding trial. The *in vitro* experiment evaluated flaxseed oil, BSFL oil and a 1:1 blend at two inclusion levels (400 and 800 mg for the single oils; 800 and 1600 mg for the blend). BSFL oil was selected to allow a direct comparison of lipid sources differing in fatty acid composition while minimizing the influence of non-lipid components. The blend doses were set at twice the corresponding single-oil doses so that, in the 1:1 blend, each oil contributed an amount equivalent to the corresponding single-oil treatment. The 1:1 blend was used as a balanced screening formulation to determine whether the combined oils adversely affected rumen fermentation and to identify a total lipid dose that did not markedly disrupt the main fermentation products. The *in vitro* phase was therefore intended to support fermentation-based dose selection rather than to determine the final blend ratio. The *in vivo* trial compared an unsupplemented control diet with 30 g/day flaxseed oil, 30 g/day BSFL oil or a 30 g/day 2:1 flaxseed-to-BSFL oil blend administered for 21 days before expected parturition. The 2:1 flaxseed-to-BSFL ratio used *in vivo* was selected to reduce the contribution of the lauric-acid-rich BSFL oil while retaining both lipid sources in the blend. This ratio was not tested directly *in vitro* and therefore represents an extrapolation from the screening phase rather than a quantitatively optimized formulation.

### 4.2. Ethics Approval and Animal Welfare

All experimental procedures involving the ewes were approved by the Local Ethical Committee for Animal Experimentation in Wrocław (Resolution No. 064/2023/P1 of 6 December 2023). Animals were maintained under conditions consistent with Directive 2010/63/EU on the protection of animals used for scientific purposes [42]. The animal-handling team included a researcher with a PhD in animal science and experienced veterinary practitioners. Rumen contents used for the *in vitro* experiment were collected during procedures approved by the Local Ethical Committee for Animal Experimentation (Protocol No. 058/2024 of 4 December 2024).

### 4.3. In Vitro Fermentation Experiment

#### 4.3.1. Donor Animals, Inoculum and Experimental Design

The experiment was conducted at the Research and Teaching Station of the Wrocław University of Environmental and Life Sciences in Swojczyce, Poland. Rumen contents were collected after the morning feeding from three non-lactating, non-pregnant, rumen-cannulated Holstein-Friesian cows (8 ± 1 years; 500 ± 50 kg) maintained under the same housing and feeding conditions. The cows were maintained as rumen-fluid donors and served solely as the source of inoculum for the *in vitro* fermentation experiment. The pooled bovine inoculum was used for qualitative screening, and direct extrapolation of the *in vitro* findings to sheep should therefore be made with caution. The cows received hay and hay silage ad libitum and had free access to water. Approximately 1 L of rumen contents was collected in total, pooled across cows, transferred to a pre-warmed insulated flask and transported to the laboratory within 30 min.

Rumen fluid was filtered through four layers of cheesecloth and combined with a fermentation buffer prepared according to the ANKOM *in vitro* procedure described by Holden [43], at a 1:4 (*v*/*v*) ratio (75 mL rumen fluid and 300 mL buffer; final pH 6.80). Each 500-mL fermentation vessel contained 1 g substrate (0.5 g hay and 0.5 g wheat grain) in an ANKOM F57 filter bag (ANKOM Technology, Macedon, NY, USA) and the assigned lipid treatment in a separate closed F57 bag. Vessels were flushed with CO_2_, incubated at 39 °C and continuously agitated. The entire *in vitro* experiment was conducted in three independent runs on three separate dates. Each treatment was represented by one fermentation vessel in each run, yielding three independent vessel replicates per treatment (n = 3).

#### 4.3.2. Sampling and Fermentation Measurements

Samples of the produced gas were collected 4 and 24 h after the commencement of the incubation process using gas-tight Hamilton syringes (Hamilton Company, Reno, NV, USA) (injection volume: 600 µL). Immediately following the collection, the gas sample was analyzed using a Shimadzu GC-2030 instrument equipped with a flame ionization detector (FID) and an SH-Q-BOND capillary column (30 m × 0.32 mm × 10 µm; Shimadzu Corporation, Kyoto, Japan). The injector (SPL) temperature was maintained at 240 °C and operated in split mode (1:10). Helium was used as the carrier gas with a linear velocity of 35.0 cm s^−1^. The oven temperature program started at 40 °C for 2.5 min, then increased at a rate of 35 °C min^−1^ to 200 °C where it was held for 2.0 min (total run time: 9.07 min). The FID was maintained at 240 °C with gas flow rates of 32.0 mL min^−1^ for H_2_, 200.0 mL min^−1^ for air, and 24.0 mL min^−1^ for He makeup gas.

Fermentation fluid was sampled at 4 and 24 h. Aliquots for volatile fatty acid and ammonia analyses were acidified with 0.5 mL 98% H_2_SO_4_ (Chempur, Piekary Śląskie, Poland) per 10 mL sample, and 50-mL aliquots were retained for long-chain fatty acid analysis. The measured outcomes were pH, ammonia nitrogen, volatile fatty acid concentrations, methane concentration and the fatty acid profile of the incubation fluid. Gas and fermentation fluid samples collected at 4 and 24 h originated from the same fermentation vessels.

To determine ammonia concentrations, 1 mL of each acidified sample underwent centrifugation (14,500× *g*, 4 min) to eliminate solid fractions. Subsequently, a 1:9 dilution of the supernatant in deionized water was prepared to ensure absorbance remained within the limits of the photometer. The analysis was conducted with a portable colorimeter (HI-97733, Hanna Instruments, Smithfield, RI, USA) according to the manufacturer’s protocol, applying the Nessler method (ASTM D1426-92) [44] using the manufacturer’s reagents (Hanna Instruments, Smithfield, RI, USA) [45]. The device’s integrated calibration curve allowed for the automatic generation of final concentration values.

### 4.4. In Vivo Experimental Procedures

#### 4.4.1. Animals, Allocation and Dietary Treatments

Thirty-two pregnant Olkuska ewes were randomly allocated to four treatment groups (n = 8 per group): control, flaxseed oil, BSFL oil and a 2:1 flaxseed-to-BSFL oil blend. Sample size was determined before the start of the experiment using an a priori power analysis performed in Statistica 13.0 (TIBCO Software Inc., Palo Alto, CA, USA) for a one-way analysis of variance comparing four independent treatment groups. Assuming a significance level of α = 0.05 and a statistical power of 80%, the analysis indicated a minimum total sample size of 31 ewes. The final sample size was increased to 32 to allow equal allocation of eight ewes to each treatment group. At the start of the study, the animals were 3 to 5 years old, had a mean body weight of 65 ± 5 kg, and had reached the late gestation phase. Indoor housing was provided at the Research and Teaching Station of the Wrocław University of Environmental and Life Sciences (Swojczyce, Poland) throughout the periparturient period. Standard environmental conditions were maintained, with the flock given unrestricted access to a basal diet and fresh water. The basal diet consisted of meadow hay provided ad libitum and a custom-made concentrate mixture supplied at 0.5 kg per animal twice daily. Based on prior chemical analyses, the dry matter of the meadow hay contained approximately 8.98% crude protein (CP), 59.85% neutral detergent fiber (NDF), and 1.03% crude fat (ether extract). The concentrate mixture, serving as the primary energy and protein source, contained approximately 16.96% CP, 33.79% NDF, and 9.61% crude fat. The complete GC–MS fatty acid profiles of the basal diet components (meadow hay and concentrate mixture) used in the present experiment have been reported previously [46].

Pregnancy and litter size were confirmed by transabdominal ultrasonography. Eligible ewes were clinically healthy and carried twins or triplets. Ewes with singleton pregnancies, more than three fetuses, unconfirmed pregnancy or clinical abnormalities were excluded before randomization. No animals were excluded after allocation, and all 32 ewes were included in the reported analyses.

The lipid sources were cold-pressed flaxseed oil (LinumVital^®^, Oleofarm, Wrocław, Poland) and BSFL oil (Grinsect/BSF Systems Kft., Hódmezővásárhely, Hungary). Supplements were administered individually at 30 g/day for 21 days before expected parturition. Ewes in the blend group received 20 g/day flaxseed oil and 10 g/day BSFL oil. Oils were mixed into the afternoon concentrate allowance, and no feed refusals were observed. The control group received the same basal feeding programme without supplemental oil. The protocol did not include an energy-matched control supplement, which limits attribution of any response specifically to fatty acid source rather than additional dietary energy.

The assessed *in vivo* outcomes were colostrum gross composition and fatty acid profile, and maternal serum markers of energy and lipid metabolism, protein status, and hepatic and renal function. Colostrum fat concentration was the principal compositional outcome. Adverse events and clinical signs were monitored throughout the study; none required treatment withdrawal.

No euthanasia or terminal procedure was performed. Ewes remained under veterinary supervision and returned to the home flock after completion of the study.

#### 4.4.2. Colostrum Sampling

Colostrum was collected from both teats as soon as possible after lambing and before routine feeding of the lambs. After discarding the first streams, approximately 30–50 mL was hand-milked into sterile tubes. One sample was obtained from each ewe (n = 8 per group; 32 samples in total), chilled immediately and frozen until analysis.

#### 4.4.3. Blood Sampling

Blood samples were collected from all experimental ewes at four time points: before the beginning of dietary supplementation (0 d; baseline), after 21 days of prepartum supplementation, and at 7 and 21 days postpartum. Blood samples were collected in the morning, before the morning feeding. Approximately 4 mL of blood was collected from each ewe by jugular venipuncture into sterile serum tubes containing a clot activator (SARSTEDT, Nümbrecht, Germany). Samples were centrifuged at 3000× *g* for 10 min at room temperature within 2 h of collection. The obtained serum was transferred into Eppendorf tubes (Eppendorf SE, Hamburg, Germany) and stored at −20 °C until biochemical analysis.

### 4.5. Laboratory Analyses

#### 4.5.1. Volatile Fatty Acids

To determine volatile fatty acids (VFAs), 1 mL of each incubation sample was transferred into 2 mL Eppendorf tubes. Each sample was spiked with 0.5 mg of heptanoic acid (C7; Sigma-Aldrich, Merck KGaA, Darmstadt, Germany) to serve as an internal standard. Subsequently, 0.8 mL of diethyl ether (Supelco, Merck KGaA, Darmstadt, Germany) was added, and the mixture was vigorously homogenized using a V-1 plus vortex mixer (Biosan SIA, Riga, Latvia) for 30 s. The samples were then centrifuged at 14,000× *g* for 4 min in a tabletop centrifuge. The resulting organic phase was separated and purified by filtration through a layer of cotton and celite. The remaining aqueous phase was subjected to a second extraction with an additional 0.8 mL of diethyl ether. Following vortexing (30 s) and centrifugation (14,000× *g*, 4 min), this second extract was filtered in the same manner. The combined ether extracts were transferred into chromatography vials prior to instrumental analysis. The determination of VFAs was performed using a Shimadzu GCMS QP 2020 system (Shimadzu Corporation, Kyoto, Japan). Data acquisition and processing were performed using GCMSsolution software, version 4.20 (Shimadzu Corporation, Kyoto, Japan). Chromatographic separation was achieved using a Zebron ZB-FAME capillary column (60 m × 0.25 mm × 0.20 μm; Phenomenex, Torrance, CA, USA). The injection port was heated to 260 °C and operated with a split ratio of 50:1. The thermal gradient of the oven was initiated at 80 °C with a 1-min hold, followed by a heating ramp of 7 °C min^−1^ until reaching a final temperature of 200 °C, which was maintained for an additional 2 min. Helium acted as the carrier gas at a constant flow rate of 1.80 mL min^−1^. Mass spectrometry parameters included a scan range of *m*/*z* 42 to 300 and an acquisition rate of 1 scan s^−1^. Quantitative analysis of individual VFAs was conducted by referencing calibration curves generated from analytical reference standards (Sigma-Aldrich, Merck KGaA, Darmstadt, Germany).

#### 4.5.2. Long-Chain Fatty Acids

To determine long-chain fatty acids (LCFAs) in fermentation-fluid samples, incubation fluid was collected in 50-mL conical polypropylene tubes (Chemland, Stargard, Poland) and aliquoted into 10 mL portions in 15-mL tubes (Chemland, Stargard, Poland). Each sample was spiked with 0.05 mg of nonadecanoic acid (C19:0; Sigma-Aldrich, Merck KGaA, Darmstadt, Germany) to serve as an internal standard. The sample was extracted twice with 5 mL of a chloroform–methanol mixture (2:1, *v*/*v*, Chempur, Piekary Śląskie, Poland). After each addition, the sample was shaken and centrifuged. The organic (chloroform) phases were combined and evaporated to dryness. Two millilitres of a 1 M potassium hydroxide solution in methanol (Chempur, Piekary Śląskie, Poland) were added to a round-bottom flask. The mixture was then heated in a heating basket (80 °C) for three minutes with the lid on. Then, 1.5 mL of BF_3_ solution (Sigma-Aldrich, Merck KGaA, Darmstadt, Germany) was added, after which the mixture was heated again (80 °C) for three minutes. A 1 mL volume of distilled water and 1 mL of aqueous NaCl solution (Chempur, Piekary Śląskie, Poland) were added to cool the reaction mixture. A 5 mL volume of cyclohexane (Chempur, Piekary Śląskie, Poland) was added, after which the samples were shaken vigorously. The mixture was transferred to 15-mL conical tubes and mixed on a vortex mixer for an additional 30 s. It was then centrifuged for four minutes in a tabletop centrifuge at 14,500× *g*. The upper cyclohexane phase was collected and filtered through a cotton and celite (Sigma-Aldrich, Merck KGaA, Darmstadt, Germany) layer before being transferred to an evaporation dish with a pipette. The solvent was evaporated to dryness, after which 300 µL of cyclohexane was added. The vessel was thoroughly rinsed with cyclohexane, and the resulting solution was transferred to a glass insert.

For the analysis of LCFAs, a 10 mL aliquot of each colostrum sample was transferred into 15 mL conical centrifuge tubes and centrifuged for 15 min at 14,500× *g* in a tabletop centrifuge. The separated fat fraction was collected and transferred into 2 mL microcentrifuge tubes. Three 30-mg aliquots of the extracted fat were weighed into separate 20-mL glass vials. Two milliliters of 1 M KOH in methanol and 0.5 mg of the internal standard (C19:0) were added to each sample. The prepared mixture was heated at 80 °C for 10 min on a magnetic hotplate stirrer. Next, 1.5 mL of BF_3_ was added, and the samples were heated at 80 °C in closed vials for an additional 3 min. For rapid cooling, 1 mL of distilled water and 1 mL of aqueous NaCl solution were added. Cyclohexane (5 mL) was then introduced, and the samples were vigorously shaken for 30 s. The mixture was transferred into 15 mL conical centrifuge tubes, vortexed for another 30 s, and centrifuged for 4 min in a benchtop centrifuge (14,000× *g*). The upper cyclohexane phase was collected, filtered through a celite bed, and transferred via pipette into an evaporation vessel, where the liquid phase was completely evaporated to dryness. Following total evaporation, 1 mL of cyclohexane was added to thoroughly rinse the bottom of the vessel, and then the resulting solution was transferred into a chromatography vial and analyzed.

LCFAs derived from both inoculum-fermentation samples and fat were analyzed using the same Shimadzu GCMS QP 2020 system (Shimadzu, Kyoto, Japan) as described for VFA determination. Chromatographic separation was carried out on a Zebron ZB-FAME capillary column (60 m × 0.25 mm × 0.20 μm; Phenomenex, Torrance, CA, USA). The injection port temperature was set to 280 °C. The split ratio was adjusted depending on the sample type, i.e., 1:10 for inoculum-fermentation samples and 1:80 for fat samples. The oven temperature program was initiated at 80 °C (hold for 2 min), followed by a ramp of 3 °C min^−1^ to 180 °C, then 8 °C min^−1^ to 240 °C with a final hold of 4 min. Helium was used as the carrier gas at a constant column flow of 1.80 mL min^−1^. Mass spectrometric detection was performed in selected ion monitoring (SIM) mode for inoculum- fermentation samples (ions: 55, 74, 79, 81, 87, and 143 *m*/*z*), whereas fat samples were analyzed in scan mode over the range of *m*/*z* 40–400 at an acquisition rate of 3 scans s^−1^. The ion source and interface temperatures were maintained at 220 °C and 250 °C, respectively.

Each fatty acid was expressed as a percentage of total identified fatty acids. Fatty acid groups and lipid quality indices were calculated from individual fatty acid proportions.

#### 4.5.3. Colostrum Composition Analysis

The basic chemical composition of sheep colostrum, including fat, protein, lactose, solids-not-fat, mineral salts, and density, was determined using a FARM ECO ultrasonic milk analyzer (Lactoscan, Milkotronic Ltd., Nova Zagora, Bulgaria; distributed by GAP Food Additives, Łopuszna, Poland). Prior to the study, the analyzer was validated for the analysis of ovine colostrum. Prior to the analysis, the colostrum samples were warmed to 40 °C in a water bath and gently mixed to ensure homogenization, in accordance with the manufacturer’s instructions.

#### 4.5.4. Blood Biochemical Analyses

Serum biochemical parameters were measured using an automated clinical chemistry analyzer (Pentra 400, Horiba ABX Diagnostics, Montpellier, France). Only parameters presented in the results tables were included in the analysis. Glucose concentration was determined using the glucose oxidase method (Horiba ABX, Montpellier, France). Non-esterified fatty acids (NEFA), triglycerides (TG), and total cholesterol (TC) were analyzed using enzymatic assays, with Randox Laboratories (Crumlin, County Antrim, UK) reagents used for NEFA and Horiba ABX reagents used for TG and TC. High-density lipoprotein cholesterol (HDL-C) and low-density lipoprotein cholesterol (LDL-C) were quantified colorimetrically using Horiba ABX reagents.

Activities of aspartate aminotransferase (AST), alanine aminotransferase (ALT), gamma-glutamyl transferase (GGT), and alkaline phosphatase (ALP) were assessed using kinetic assays with Horiba ABX reagents. Total bilirubin (TBIL), total protein (TP), albumin (ALB), and creatinine (CREA) were determined using colorimetric methods, while blood urea nitrogen (BUN) was determined using a UV enzymatic method; all parameters were measured using Horiba ABX reagents, according to the manufacturer’s instructions.

Total antioxidant status (TAS) in serum was evaluated colorimetrically using an ABTS-based assay (2,2′-azino-bis[3-ethylbenzothiazoline-6-sulfonic acid]) in the presence of peroxidase and H_2_O_2_, using Randox reagents. The relatively stable blue-green complex was measured at a wavelength of 600 nm on a biochemical analyzer (Pentra 400, Horiba ABX Diagnostics, France).

#### 4.5.5. Statistical Analysis

Analyses were performed in Statistica 13.0 (TIBCO Software Inc., Palo Alto, CA, USA). Distributional assumptions and variance homogeneity were evaluated using Shapiro–Wilk and Levene tests. Results are reported as raw or adjusted means with SEM, as specified in the table footnotes. All tests were two-sided and *p* < 0.05 was considered statistically significant. Results with 0.05 ≤ *p* < 0.10 are described as numerical tendencies only and are not interpreted as evidence of an effect.

For the *in vitro* experiment, the three runs conducted on separate dates provided the independent replicates. Each treatment was represented by one fermentation vessel per run; therefore, the fermentation vessel was the experimental unit and n = 3 per treatment. Fermentation variables were analyzed using a repeated-measures model, with treatment as the between-vessel fixed effect, incubation time (4 and 24 h) as the within-vessel repeated factor, and treatment × incubation time as the interaction term. The fermentation vessel was treated as the subject for repeated measurements. Pairwise comparisons among treatments within each time point were Tukey-adjusted. Fatty acid profiles were analyzed separately at 4 and 24 h using the Kruskal–Wallis test. When a significant treatment effect was detected, multiple comparisons of mean ranks with Bonferroni adjustment were used for post hoc comparisons. Analytical replicate measurements, where performed, were averaged and were not treated as independent experimental units.

For colostrum chemical composition and fatty acid profile, treatment was included as the fixed effect and the ewe was the experimental unit. Colostrum was collected only once, immediately after lambing (0 h postpartum), and each ewe contributed one observation to the statistical model. Treatment effects were evaluated using one-way ANOVA when the assumptions of normality and homogeneity of variance were met. When variances were heterogeneous, Welch’s ANOVA was applied. Pairwise comparisons among treatments were performed using the Tukey–Kramer test, whereas Dunnett’s test was used when the primary comparison was against the control group. If the assumptions for parametric analysis were not met, the Kruskal–Wallis test was applied, followed by multiple comparisons of mean ranks.

Blood biochemical variables were analyzed using repeated-measures analysis of covariance with a multivariate approach. Treatment, time, and treatment × time were included as fixed effects. The corresponding baseline value was included as a covariate. The ewe was the experimental unit and the subject for repeated measurements. Measurements obtained at the end of supplementation and at 7 and 21 days postpartum were treated as repeated post-baseline outcomes, whereas the corresponding pre-supplementation value obtained at 0 d was included as a covariate. The treatment effect was evaluated for the baseline-adjusted mean across the three post-baseline sampling times using an F test, whereas time and treatment × time were evaluated using Pillai’s trace. BHB, TG, HDL-C, LDL-C, AST, GGT, ALP, TBIL, and BUN were natural-log transformed before analysis. For GGT, treatment × baseline terms were additionally included because the homogeneity-of-regression-slopes assumption was not met. Adjusted least-squares means are presented on the original scale; estimates for natural-log-transformed variables were back-transformed, and their standard errors were obtained using the delta method. Pairwise comparisons were adjusted using Holm’s method. Each biochemical parameter was analyzed as a separate dependent variable using the same model structure.

Missing observations, if any, were not imputed. The ewe was the experimental unit for all *in vivo* analyses.

## 5. Conclusions

Low-α-linolenic/high-linoleic flaxseed oil and lauric-acid-rich BSFL oil acted as compositionally distinct alternative lipid feed materials. *In vitro*, they produced source- and dose-dependent changes in methane, ammonia, and fatty acid profiles, while total VFA concentration remained unchanged and the relative fermentation pattern shifted after 24 h. In prolific ewes, a higher colostrum fat concentration was observed in the 2:1 flaxseed-to-BSFL oil blend group, together with differences in selected serum metabolic variables and minor colostrum fatty acids. Serum TAS remained unchanged, providing no evidence of a treatment-related effect on this marker of circulating antioxidant capacity under the conditions studied. These findings warrant further validation under commercial conditions before practical feeding recommendations can be made.

## Figures and Tables

**Table 1:** *In vitro* fermentation characteristics after lipid supplementation.

Time	Treatments							SEM	*p*-Value
	Control	Flaxseed Oil		BSFL Oil		Mix ^1^ Oil			
		400 mg	800 mg	400 mg	800 mg	800 mg	1600 mg		
pH									
4 h	6.64 ^b^	6.70 ^a^	6.69 ^a^	6.67 ^ab^	6.71 ^a^	6.69 ^a^	6.69 ^a^	0.009	0.001
24 h	6.54 ^ab^	6.59 ^a^	6.58 ^ab^	6.53 ^b^	6.55 ^ab^	6.57 ^ab^	6.55 ^ab^	0.012	0.022
NH_3_-N, mg dL^−1^									
4 h	12.4 ^d^	11.4 ^e^	11.9 ^e^	12.6 ^d^	15.9 ^a^	15.2 ^b^	13.6 ^c^	0.102	<0.001
24 h	23.0 ^d^	23.4 ^d^	24.0 ^c^	25.4 ^a^	25.4 ^a^	24.0 ^c^	24.6 ^b^	0.085	<0.001
Total VFA, mg mL^−1^									
4 h	1.74	2.22	2.20	1.62	1.84	1.97	1.93	0.327	0.821
24 h	2.50	3.06	2.39	1.80	2.94	2.86	2.30	0.466	0.525
Individual VFA, [% of total VFA]									
Acetate									
4 h	56.90	59.52	59.49	56.57	56.69	57.26	57.98	1.550	0.673
24 h	57.87	56.48	56.44	55.41	54.56	53.57	56.20	1.255	0.332
Propionate									
4 h	22.01	21.90	21.73	21.81	21.59	22.50	21.68	0.192	0.075
24 h	23.13 ^bc^	24.88 ^a^	23.79 ^ab^	22.18 ^c^	25.12 ^a^	24.87 ^a^	23.02 ^bc^	0.302	<0.001
Butyrate									
4 h	21.09	18.59	18.78	21.63	21.72	20.24	20.34	1.502	0.646
24 h	19.00	18.65	19.77	22.41	20.31	21.56	20.78	1.384	0.489
Acetate to propionate ratio									
4 h	2.59	2.72	2.74	2.60	2.63	2.54	2.68	0.081	0.599
24 h	2.50 ^a^	2.28 ^ab^	2.37 ^ab^	2.50 ^a^	2.17 ^b^	2.15 ^b^	2.44 ^a^	0.052	0.001
Methane, mL L^−1^									
4 h	3.75 ^c^	2.44 ^d^	1.55 ^e^	3.41 ^c^	6.99 ^a^	4.98 ^b^	2.79 ^d^	0.096	<0.001
24 h	13.72 ^b^	10.18 ^c^	7.42 ^d^	11.26 ^c^	22.10 ^a^	15.27 ^b^	10.64 ^c^	0.368	<0.001

^1^ Mix oil consisted of flaxseed oil and BSFL oil at a 1:1 ratio. Values are presented as means with SEM. *p*-values refer to the overall treatment effect within each incubation time. Within each incubation time, means within the same row that do not share a common superscript letters (a–e) differ significantly according to Tukey-adjusted pairwise comparisons (*p* < 0.05), whereas means sharing at least one superscript letter do not differ significantly. NH_3_-N, ammonia nitrogen; VFA, volatile fatty acids.

**Table 2:** *In vitro* fatty acid profile after 4 h [% of total identified fatty acids].

	Treatments							SEM	*p*-Value
	4 h								
	Control	Flaxseed Oil		BSFL Oil		Mix ^1^ Oil			
		Dose [mg]							
		400	800	400	800	800	1600		
C12:0	1.61 ^ab^	0.70 ^a^	3.10 ^ab^	30.86 ^ab^	44.60 ^b^	39.07 ^ab^	32.17 ^ab^	3.97	0.003
C13:0	0.33 ^a^	0.23 ^ab^	0.15 ^ab^	0.09 ^ab^	0.07 ^ab^	0.12 ^ab^	0.06 ^b^	0.02	0.003
C14:0	1.88 ^ab^	1.70 ^a^	5.79 ^ab^	6.56 ^ab^	9.53 ^b^	7.25 ^ab^	8.37 ^ab^	0.63	0.003
*iso*-C15:0	0.89 ^a^	0.72 ^ab^	0.43 ^ab^	0.30 ^ab^	0.27 ^ab^	0.34 ^ab^	0.22 ^b^	0.05	0.003
*anteiso*-C15:0	0.81 ^a^	0.63 ^ab^	0.40 ^ab^	0.27 ^ab^	0.21 ^ab^	0.29 ^ab^	0.16 ^b^	0.07	0.003
C15:0	2.20 ^a^	1.46 ^ab^	1.36 ^ab^	0.61 ^ab^	0.49 ^ab^	0.48 ^ab^	0.48 ^b^	0.14	0.003
*iso*-C16:0	0.55 ^a^	0.38 ^ab^	0.32 ^ab^	0.16 ^ab^	0.07 ^b^	0.18 ^ab^	0.10 ^ab^	0.04	0.003
C16:0	37.92 ^ab^	30.95 ^ab^	39.89 ^a^	24.95 ^ab^	22.70 ^b^	25.17 ^ab^	23.33 ^ab^	1.48	0.003
*anteiso*-C16:0	0.07 ^ab^	0.06 ^ab^	0.18 ^a^	0.05 ^ab^	0.06 ^ab^	0.05 ^b^	0.06 ^ab^	0.01	0.003
C16:1 (n-7)	0.02 ^a^	0.06 ^ab^	0.17 ^ab^	0.49 ^ab^	0.78 ^b^	0.53 ^ab^	0.77 ^ab^	0.07	0.003
*iso*-C17:0	1.09 ^a^	0.94 ^ab^	1.03 ^a^	0.41 ^ab^	0.28 ^ab^	0.68 ^ab^	0.19 ^b^	0.08	0.004
*anteiso*-C17:0	0.32 ^a^	0.33 ^a^	0.21 ^ab^	0.16 ^ab^	0.10 ^ab^	0.13 ^ab^	0.08 ^b^	0.02	0.004
C17:0	1.08 ^ab^	0.88 ^ab^	1.13 ^a^	0.71 ^ab^	0.64 ^b^	0.71 ^ab^	0.66 ^ab^	0.04	0.003
C17:1	0.04 ^ab^	0.06 ^ab^	0.14 ^a^	0.03 ^ab^	0.02 ^b^	0.07 ^ab^	0.02 ^b^	0.01	0.004
*iso*-C18:0	0.11 ^ab^	0.12 ^a^	0.05 ^ab^	0.03 ^ab^	0.04 ^ab^	0.02 ^b^	0.04 ^ab^	0.02	0.004
C18:0	48.13 ^a^	35.77 ^ab^	26.43 ^ab^	19.07 ^ab^	12.75 ^b^	19.13 ^ab^	14.57 ^ab^	2.65	0.003
C18:1, *trans*-11	1.01 ^a^	6.28 ^ab^	7.97 ^b^	4.93 ^ab^	3.71 ^ab^	2.56 ^ab^	5.57 ^ab^	0.49	0.003
C18:1, *cis*-9	0.21 ^ab^	0.27 ^ab^	0.87 ^a^	0.15 ^b^	0.16 ^ab^	0.42 ^ab^	0.34 ^ab^	0.05	0.003
C18:1 n-7	0.07 ^ab^	0.08 ^ab^	0.19 ^a^	0.04 ^ab^	0.02 ^b^	0.03 ^ab^	0.09 ^ab^	0.01	0.003
C18:2 n-6	0.27 ^a^	10.21 ^b^	5.63 ^ab^	6.00 ^ab^	2.62 ^ab^	1.84 ^ab^	7.79 ^ab^	0.73	0.003
C18:3 n-3 (ALA)	0.18 ^a^	6.43 ^b^	3.54 ^ab^	3.17 ^ab^	0.38 ^ab^	0.32 ^ab^	4.13 ^ab^	0.49	0.003
CLA	1.04 ^a^	0.91 ^ab^	0.67 ^ab^	0.47 ^ab^	0.38 ^b^	0.48 ^ab^	0.41 ^ab^	0.05	0.003
Others	0.19 ^ab^	0.83 ^a^	0.34 ^ab^	0.47 ^ab^	0.12 ^ab^	0.12 ^b^	0.39 ^ab^	0.05	0.003
SFA	96.99 ^a^	74.87 ^b^	80.47 ^ab^	84.23 ^ab^	91.79 ^ab^	93.62 ^ab^	80.48 ^ab^	1.69	0.003
MUFA	1.34 ^a^	6.74 ^ab^	9.35 ^b^	5.65 ^ab^	4.70 ^ab^	3.60 ^ab^	6.79 ^ab^	0.53	0.003
PUFA	1.49 ^a^	17.55 ^b^	9.85 ^ab^	9.64 ^ab^	3.38 ^ab^	2.65 ^ab^	12.34 ^ab^	1.22	0.003
UFA	2.83 ^a^	24.30 ^b^	19.19 ^ab^	15.30 ^ab^	8.08 ^ab^	6.25 ^ab^	19.12 ^ab^	1.64	0.003
OCFA	3.64 ^a^	2.63 ^ab^	2.78 ^ab^	1.44 ^ab^	1.22 ^b^	1.38 ^ab^	1.22 ^b^	0.20	0.003
BCFA	3.84 ^a^	3.18 ^ab^	2.62 ^ab^	1.39 ^ab^	1.03 ^ab^	1.69 ^ab^	0.84 ^b^	0.23	0.003
OBCFA	7.48 ^a^	5.81 ^ab^	5.40 ^ab^	2.83 ^ab^	2.25 ^ab^	3.07 ^ab^	2.06 ^b^	0.42	0.003
n-6 PUFA	0.27 ^a^	10.21 ^b^	5.63 ^ab^	6.00 ^ab^	2.62 ^ab^	1.84 ^ab^	7.79 ^ab^	0.73	0.003
n-3 PUFA	0.18 ^a^	6.43 ^b^	3.54 ^ab^	3.17 ^ab^	0.38 ^ab^	0.32 ^ab^	4.13 ^ab^	0.49	0.003
n-6/n-3	1.50 ^a^	1.59 ^ab^	1.59 ^ab^	1.90 ^ab^	6.81 ^b^	5.73 ^ab^	1.88 ^ab^	0.47	0.003

^1^ Mix oil-the blend consisted of flaxseed oil and BSFL oil at a 1:1 ratio. Values are presented as arithmetic means (n = 3 per treatment). SEM, standard error of the mean. *p*-values were obtained using the Kruskal–Wallis test. Means within a row that do not share a common superscript letters (a,b) differ significantly according to Bonferroni-adjusted multiple comparisons of mean ranks (*p* < 0.05); ALA = α-linolenic acid; SFA = sum of the individual saturated fatty acids; UFA = sum of the individual unsaturated fatty acids; MUFA = sum of the individual monounsaturated fatty acids; PUFA = sum of the individual polyunsaturated fatty acids; OCFA = odd-chain fatty acids; BCFA = branched-chain fatty acids, sum of iso- and anteiso-FA; OBCFA = odd- and branched-chain fatty acids, sum of odd-, iso-, and anteiso-FA; n-3 PUFA and n-6 PUFA = sum of individual n-3 and n-6 fatty acids, respectively.

**Table 3:** *In vitro* fatty acid profile after 24 h [% of total identified fatty acids].

	Treatments							SEM	*p*-Value
	24 h								
	Control	Flaxseed Oil		BSFL Oil		Mix ^1^ Oil			
		Dose [mg]							
		400	800	400	800	800	1600		
C12:0	2.16 ^ab^	0.76 ^ab^	0.74 ^a^	36.07 ^ab^	42.54 ^b^	32.44 ^ab^	37.55 ^ab^	4.02	0.003
C13:0	0.50 ^a^	0.23 ^ab^	0.27 ^ab^	0.23 ^ab^	0.09 ^ab^	0.08 ^ab^	0.06 ^b^	0.03	0.003
C14:0	2.24 ^ab^	1.48 ^ab^	1.36 ^a^	6.44 ^ab^	6.76 ^ab^	5.81 ^ab^	7.77 ^b^	0.57	0.003
*iso*-C15:0	1.03 ^a^	0.57 ^ab^	0.72 ^ab^	0.46 ^ab^	0.27 ^ab^	0.27 ^ab^	0.18 ^b^	0.06	0.003
*anteiso*-C15:0	1.34 ^a^	0.63 ^ab^	0.87 ^ab^	0.35 ^ab^	0.30 ^ab^	0.34 ^ab^	0.21 ^b^	0.08	0.004
C15:0	2.95 ^a^	1.43 ^ab^	1.71 ^ab^	0.97 ^ab^	0.64 ^ab^	0.61 ^ab^	0.43 ^b^	0.18	0.003
*iso*-C16:0	0.74 ^a^	0.33 ^ab^	0.46 ^ab^	0.19 ^ab^	0.11 ^ab^	0.10 ^ab^	0.08 ^b^	0.05	0.003
C16:0	35.97 ^a^	24.62 ^ab^	30.22 ^ab^	24.99 ^ab^	25.12 ^ab^	21.33 ^ab^	20.50 ^b^	1.11	0.003
*anteiso*-C16:0	0.05 ^ab^	0.02 ^ab^	0.05 ^ab^	0.29 ^a^	0.16 ^ab^	0.02 ^b^	0.06 ^ab^	0.02	0.003
C16:1 (n-7)	0.05 ^ab^	0.05 ^ab^	0.04 ^a^	0.32 ^ab^	0.92 ^b^	0.27 ^ab^	0.62 ^ab^	0.07	0.003
*iso*-C17:0	0.95 ^a^	0.57 ^ab^	0.82 ^ab^	0.55 ^ab^	0.26 ^ab^	0.36 ^ab^	0.22 ^b^	0.06	0.003
*anteiso*-C17:0	0.55 ^a^	0.30 ^ab^	0.41 ^ab^	0.21 ^ab^	0.12 ^ab^	0.12 ^ab^	0.07 ^b^	0.04	0.003
C17:0	2.03 ^a^	1.30 ^ab^	1.59 ^ab^	1.32 ^ab^	1.32 ^ab^	1.12 ^ab^	1.08 ^b^	0.07	0.003
C17:1	0.07 ^ab^	0.04 ^ab^	0.04 ^ab^	1.00 ^a^	0.03 ^ab^	0.02 ^ab^	0.02 ^b^	0.08	0.003
*iso*-C18:0	0.11 ^ab^	0.28 ^a^	0.06 ^ab^	0.06 ^ab^	0.03 ^ab^	0.04 ^ab^	0.03 ^b^	0.02	0.003
C18:0	46.71 ^ab^	39.44 ^ab^	47.67 ^a^	18.31 ^ab^	13.60 ^b^	23.50 ^ab^	14.16 ^ab^	3.13	0.003
C18:1, *trans*-11	0.58 ^a^	11.31 ^b^	3.54 ^ab^	3.19 ^ab^	4.93 ^ab^	4.81 ^ab^	5.83 ^ab^	0.68	0.003
C18:1, *cis*-9	0.17 ^ab^	1.26 ^ab^	0.65 ^ab^	0.22 ^ab^	0.13 ^a^	3.45 ^b^	0.39 ^ab^	0.25	0.003
C18:1 n-7	0.08 ^ab^	0.42 ^a^	0.27 ^ab^	0.06 ^ab^	0.05 ^b^	0.23 ^ab^	0.08 ^ab^	0.03	0.003
C18:2 n-6	0.26 ^a^	7.81 ^b^	4.18 ^ab^	2.20 ^ab^	1.52 ^ab^	2.80 ^ab^	6.61 ^ab^	0.57	0.003
C18:3 n-3 (ALA)	0.21 ^ab^	4.76 ^a^	2.48 ^ab^	1.04 ^ab^	0.06 ^b^	1.13 ^ab^	3.39 ^ab^	0.36	0.003
CLA	1.10 ^a^	0.76 ^ab^	0.94 ^ab^	0.37 ^ab^	0.44 ^ab^	0.48 ^ab^	0.30 ^b^	0.06	0.003
Others	0.14 ^a^	1.61 ^b^	0.90 ^ab^	1.16 ^ab^	0.61 ^ab^	0.67 ^ab^	0.37 ^ab^	0.10	0.003
SFA	97.34 ^a^	71.98 ^b^	86.95 ^ab^	90.44 ^ab^	91.31 ^ab^	86.15 ^ab^	82.41 ^ab^	1.66	0.003
MUFA	0.96 ^a^	13.08 ^b^	4.55 ^ab^	4.79 ^ab^	6.06 ^ab^	8.78 ^ab^	6.92 ^ab^	0.79	0.003
PUFA	1.57 ^a^	13.33 ^b^	7.60 ^ab^	3.61 ^ab^	2.02 ^ab^	4.40 ^ab^	10.29 ^ab^	0.92	0.003
UFA	2.52 ^a^	26.41 ^b^	12.15 ^ab^	8.40 ^ab^	8.08 ^ab^	13.18 ^ab^	17.22 ^ab^	1.59	0.003
OCFA	5.56 ^a^	3.00 ^ab^	3.61 ^ab^	3.53 ^ab^	2.08 ^ab^	1.83 ^ab^	1.59 ^b^	0.29	0.003
BCFA	4.77 ^a^	2.71 ^ab^	3.39 ^ab^	2.11 ^ab^	1.25 ^ab^	1.25 ^ab^	0.85 ^b^	0.29	0.003
OBCFA	10.33 ^a^	5.71 ^ab^	7.00 ^ab^	5.64 ^ab^	3.33 ^ab^	3.09 ^ab^	2.44 ^b^	0.57	0.003
n-6 PUFA	0.26 ^a^	7.81 ^b^	4.18 ^ab^	2.20 ^ab^	1.52 ^ab^	2.80 ^ab^	6.61 ^ab^	0.57	0.003
n-3 PUFA	0.21 ^ab^	4.76 ^a^	2.48 ^ab^	1.04 ^ab^	0.06 ^b^	1.13 ^ab^	3.39 ^ab^	0.36	0.003
n-6/n-3	1.26 ^a^	1.64 ^ab^	1.69 ^ab^	2.12 ^ab^	24.53 ^b^	2.47 ^ab^	1.95 ^ab^	1.78	0.003

^1^  Mix oil-the blend consisted of flaxseed oil and BSFL oil at a 1:1 ratio. Values are presented as arithmetic means (n = 3 per treatment). SEM, standard error of the mean. *p*-values were obtained using the Kruskal–Wallis test. Means within a row that do not share a common superscript letters (a,b) differ significantly according to Bonferroni-adjusted multiple comparisons of mean ranks (*p* < 0.05); ALA = α-linolenic acid; SFA = sum of the individual saturated fatty acids; UFA = sum of the individual unsaturated fatty acids; MUFA = sum of the individual monounsaturated fatty acids; PUFA = sum of the individual polyunsaturated fatty acids; OCFA = odd-chain fatty acids; BCFA = branched-chain fatty acids, sum of iso- and anteiso-FA; OBCFA = odd- and branched-chain fatty acids, sum of odd-, iso- and anteiso-FA; n-3 PUFA and n-6 PUFA = sum of individual n-3 and n-6 fatty acids, respectively.

**Table 4:** Effect of dietary oil supplementation on the chemical composition of sheep colostrum.

Colostrum	Control	Flaxseed Oil	BSFL Oil	Mix ^1^ Oil	SEM	*p*-Value
Fat [%]	8.26	8.25	8.69	11.29 *	0.809	0.036
SNF [%]	22.64	21.53	22.75	22.41	0.986	0.802
Density [kg·m^−3^]	1075.96	1074.83	1078.61	1076.05	3.429	0.877
TP [%]	8.26	7.89	8.33	8.20	0.356	0.813
Lactose [%]	6.23	5.92	6.26	6.17	0.272	0.803
Mineral salts [%]	1.85	1.76	1.86	1.83	0.081	0.800

^1^ Mix oil-the blend consisted of flaxseed oil and BSFL oil at a 2:1 ratio. SNF—solids-not-fat; TP—total protein; SEM—pooled standard error of the mean. * Significantly different from the control group at Dunnett-adjusted *p* < 0.05.

**Table 5:** Fatty acid profile of colostrum from ewes supplemented with different oils [% of total fatty acids].

Fatty Acid	Treatments				Pooled SEM	*p*-Value
	Control	Flaxseed Oil	BSFL Oil	Mix ^1^ Oil		
C8:0	1.055	1.363	1.298	1.379	0.187	0.611
C10:0	3.292	4.349	4.084	4.631	0.626	0.495
C12:0	4.106	4.356	5.379	4.662	0.439	0.245
C13:0	0.104	0.090	0.068	0.069	0.010	0.043
C14:0	16.536	16.870	16.829	15.935	0.960	0.887
*iso*-C15:0	0.324	0.319	0.316	0.284	0.030	0.773
*anteiso*-C15:0	0.261	0.216	0.198	0.210	0.019	0.149
C15:0	1.057	0.824	0.861	0.924	0.062	0.069
*iso*-C16:0	0.227 ^a^	0.183 ^ab^	0.166 ^b^	0.174 ^ab^	0.014	0.026
C16:0	38.226	37.909	38.592	36.561	0.890	0.412
*anteiso*-C16:0	0.147 ^a^	0.086 ^b^	0.080 ^b^	0.055 ^b^	0.015	0.002
C16:1, *cis*-9	0.523	0.570	0.563	0.555	0.049	0.914
*iso*-C17:0	0.547	0.458	0.444	0.503	0.043	0.364
*anteiso*-C17:0	0.550	0.444	0.370	0.437	0.055	0.149
C17:0	1.018	1.009	1.028	0.974	0.024	0.412
C17:1, *cis*-10	0.253	0.199	0.186	0.238	0.031	0.397
*iso*-C18:0	0.117	0.097	0.088	0.096	0.016	0.654
C18:0	11.306	10.627	10.552	11.097	1.068	0.952
C18:1, *cis*-9	16.207	15.257	14.809	16.389	1.164	0.745
C18:1 n-7	0.342	0.386	0.389	0.414	0.057	0.847
C18:1 isomers	0.154	0.189	0.162	0.207	0.022	0.339
C18:2 n-6	1.882	2.175	1.757	2.082	0.185	0.381
C18:2 isomers	0.069	0.065	0.059	0.062	0.010	0.913
C18:3 n-3 (ALA)	0.480	0.535	0.415	0.557	0.036	0.048
CLA, *cis*-9 *trans*-11	0.651	0.904	0.840	0.980	0.106	0.192
C20:1 n-9	0.032	0.049	0.035	0.047	0.006	0.071
CLA isomers	0.083	0.080	0.077	0.078	0.003	0.630
C22:0	0.115	0.098	0.095	0.105	0.010	0.553
C20:4 n-6 (ARA)	0.107 ^a^	0.071 ^ab^	0.064 ^b^	0.059 ^b^	0.010	0.014
C20:5 n-3	0.057	0.055	0.052	0.057	0.005	0.859
C22:5 n-3	0.136	0.128	0.117	0.129	0.011	0.686
C22:6 n-3	0.056	0.049	0.043	0.048	0.005	0.355
**Fatty acid groups and lipid indices**						
MCFA	8.453	10.069	10.762	10.672	1.128	0.486
LCFA	91.565	89.941	89.256	89.328	1.129	0.483
SFA	78.986	79.298	80.450	78.097	1.479	0.741
MUFA	17.511	16.649	16.144	17.850	1.246	0.763
PUFA	3.520	4.062	3.424	4.053	0.289	0.267
UFA	21.031	20.712	19.568	21.903	1.479	0.745
OCFA	4.113	3.559	3.471	3.640	0.153	0.059
BCFA	2.172	1.802	1.662	1.760	0.125	0.052
OBCFA	4.605 ^a^	3.924 ^b^	3.805 ^b^	3.965 ^ab^	0.175	0.019
n-6 PUFA	1.989	2.247	1.821	2.142	0.191	0.430
n-3 PUFA	0.729	0.768	0.626	0.792	0.047	0.100
n-6/n-3	2.781	2.971	2.862	2.731	0.221	0.864
n-3/n-6	0.375	0.355	0.370	0.371	0.030	0.960
AI	5.383	5.734	6.000	4.869	0.599	0.579
TI	5.556	5.729	6.039	5.041	0.450	0.474

^1^ Mix oil–the blend consisted of flaxseed oil and BSFL oil at a 2:1 ratio. Values are presented as raw means. Means within a row that do not share a common superscript letters (a,b) differ significantly (*p* < 0.05; Tukey–Kramer test); means sharing at least one superscript letter do not differ significantly. One-way ANOVA was used for homogeneous variances (Levene’s test, *p* ≥ 0.05), whereas Welch’s ANOVA was applied when variances were heterogeneous (*p* < 0.05). SEM, pooled standard error of the mean. *p*-values were obtained using one-way ANOVA or Welch’s ANOVA, as appropriate according to the homogeneity-of-variance assumption; BSFL, black soldier fly larvae oil (*Hermetia illucens*); ALA, α-linolenic acid; ARA, arachidonic acid; MCFA, medium-chain fatty acids (C8:0–C12:0); LCFA, long-chain fatty acids (C13:0–C22:6 n-3); SFA, saturated fatty acids; UFA, unsaturated fatty acids; MUFA, monounsaturated fatty acids; PUFA, polyunsaturated fatty acids; OCFA, odd-chain fatty acids; BCFA, branched-chain fatty acids; OBCFA, odd- and branched-chain fatty acids; AI, atherogenicity index; TI, thrombogenicity index. n-6 PUFA = C18:2 n-6 + C20:4 n-6; n-3 PUFA = C18:3 n-3 + C20:5 n-3 + C22:5 n-3 + C22:6 n-3. AI = [C12:0 + (4 × C14:0) + C16:0]/UFA; TI = [C14:0 + C16:0 + C18:0]/[0.5 × MUFA + 0.5 × n-6 PUFA + 3 × n-3 PUFA + (n-3 PUFA/n-6 PUFA)].

**Table 6:** Energy metabolism and lipid profile parameters in serum of ewes across the experimental period.

Blood Parameters	Treatments				SEM	*p*-Value		
	Control	Flaxseed Oil	BSFL Oil	Mix ^1^ Oil		Treatment	Time	Treatment × Time
GLU [mmol/L]								
Baseline (0 d)	3.12	3.19	3.17	3.39	0.20			
End suppl. (21 d)	2.92 ^bAB^	3.56 ^aAB^	3.40 ^abAB^	3.21 ^abAB^	0.14	0.003	0.008	0.766
7 d postpartum	2.86 ^bB^	3.43 ^aB^	2.95 ^abB^	3.15 ^abB^	0.20			
21 d postpartum	3.17 ^bA^	3.74 ^aA^	3.42 ^abA^	3.70 ^abA^	0.16			
NEFA [mmol/L]								
Baseline (0 d)	0.38	0.29	0.33	0.44	0.10			
End suppl. (21 d)	0.41	0.38	0.43	0.50	0.06	0.262	0.047	0.405
7 d postpartum	0.38	0.33	0.51	0.55	0.09			
21 d postpartum	0.42	0.30	0.33	0.32	0.06			
BHB [mmol/L]								
Baseline (0 d)	0.27	0.26	0.25	0.22	0.04			
End suppl. (21 d)	0.42 ^B^	0.20 ^B^	0.23 ^B^	0.22 ^B^	0.08	0.042	0.001	0.191
7 d postpartum	0.42 ^A^	0.38 ^A^	0.43 ^A^	0.48 ^A^	0.07			
21 d postpartum	0.37 ^A^	0.35 ^A^	0.34 ^A^	0.48 ^A^	0.05			
TG [mmol/L]								
Baseline (0 d)	0.27	0.31	0.27	0.38	0.03			
End suppl. (21 d)	0.26 ^bA^	0.36 ^abA^	0.40 ^abA^	0.57 ^aA^	0.08	0.128	<0.001	0.014
7 d postpartum	0.13 ^B^	0.14 ^c^	0.19 ^B^	0.17 ^B^	0.02			
21 d postpartum	0.19 ^A^	0.18 ^B^	0.15 ^B^	0.15 ^B^	0.02			
TC [mmol/L]								
Baseline (0 d)	1.93	1.88	1.93	2.36	0.13			
End suppl. (21 d)	2.23 ^bA^	2.34 ^bA^	2.58 ^bA^	2.72 ^aA^	0.15	0.012	<0.001	0.536
7 d postpartum	1.68 ^bB^	1.62 ^bB^	1.60 ^bB^	1.97 ^aB^	0.15			
21 d postpartum	1.94 ^bB^	1.60 ^bB^	1.57 ^bB^	2.20 ^aB^	0.20			
HDL-C [mmol/L]								
Baseline (0 d)	1.21	1.05	1.17	1.33	0.06			
End suppl. (21 d)	1.50 ^abA^	1.44 ^bA^	1.72 ^abA^	1.73 ^aA^	0.09	0.045	<0.001	0.430
7 d postpartum	1.26 ^abc^	1.07 ^bc^	1.10 ^abc^	1.36 ^ac^	0.12			
21 d postpartum	1.57 ^abB^	1.20 ^bB^	1.24 ^abB^	1.65 ^aB^	0.18			
LDL-C [mmol/L]								
Baseline (0 d)	0.46	0.52	0.46	0.68	0.08			
End suppl. (21 d)	0.40 ^A^	0.48 ^A^	0.50 ^A^	0.53 ^A^	0.06	0.362	<0.001	0.670
7 d postpartum	0.23 ^B^	0.32 ^B^	0.27 ^B^	0.32 ^B^	0.04			
21 d postpartum	0.24 ^c^	0.21 ^c^	0.19 ^c^	0.22 ^c^	0.04			

^1^ Mix oil consists of flaxseed oil and BSFL oil in a 2:1 ratio. Baseline values are arithmetic means (n = 8 per treatment), whereas post-baseline values are baseline-adjusted least-squares means. The largest SEM among the four treatment means is presented for each sampling time. *p*-values refer to treatment, time, and treatment × time effects during the post-baseline period, with the corresponding baseline value included as a covariate. BHB, TG, HDL-C, and LDL-C were analyzed after natural-log transformation; back-transformed least-squares means are presented. When the treatment × time interaction was not significant, lowercase and uppercase superscripts indicate differences among marginal treatment and time means, respectively. When the interaction was significant, lowercase superscripts indicate treatment differences within a sampling time, and uppercase superscripts indicate time differences within a treatment (Holm-adjusted *p* < 0.05). GLU, glucose; NEFA, non-esterified fatty acids; BHB, β-hydroxybutyrate; TG, triglycerides; TC, total cholesterol; HDL-C, high-density lipoprotein cholesterol; LDL-C, low-density lipoprotein cholesterol.

**Table 7:** Liver enzyme activities and total antioxidant status in ewes across the experimental period.

Blood Parameters	Treatments				SEM	*p*-Value		
	Control	Flaxseed Oil	BSFL Oil	Mix ^1^ Oil		Treatment	Time	Treatment × Time
AST [U/L]								
Baseline (0 d)	104.39	110.85	108.30	109.93	7.73			
End suppl. (21 d)	124.95 ^c^	130.11 ^c^	118.08 ^c^	120.63 ^c^	8.41	0.432	<0.001	0.569
7 d postpartum	133.63 ^B^	150.61 ^B^	129.96 ^B^	142.53 ^B^	8.14			
21 d postpartum	150.53 ^A^	159.19 ^A^	170.08 ^A^	190.93 ^A^	18.59			
ALT [U/L]								
Baseline (0 d)	18.72	20.22	21.76	19.33	1.60			
End suppl. (21 d)	21.66 ^A^	21.89 ^A^	20.82 ^A^	20.49 ^A^	1.11	0.972	0.030	0.598
7 d postpartum	19.27 ^B^	19.02 ^B^	18.70 ^B^	19.32 ^B^	1.31			
21 d postpartum	19.70 ^AB^	18.99 ^AB^	21.94 ^AB^	21.41 ^AB^	1.44			
GGT [U/L]								
Baseline (0 d)	49.37	51.03	52.18	50.54	3.03			
End suppl. (21 d)	49.82 ^B^	56.97 ^B^	55.09 ^B^	50.25 ^c^	2.57	0.177	<0.001	0.006
7 d postpartum	66.23 ^A^	73.64 ^A^	60.66 ^AB^	65.56 ^B^	4.51			
21 d postpartum	66.95 ^bA^	74.79 ^abA^	69.39 ^bA^	98.62 ^aA^	8.55			
ALP [U/L]								
Baseline (0 d)	110.08	91.41	89.08	170.31	36.85			
End suppl. (21 d)	108.44	92.61	119.52	150.89	35.57	0.263	0.069	0.604
7 d postpartum	116.50	102.31	79.51	106.22	24.57			
21 d postpartum	157.41	130.09	91.41	212.92	64.60			
TBIL [µmol/L]								
Baseline (0 d)	2.08	1.69	1.95	1.96	0.31			
End suppl. (21 d)	1.98 ^A^	2.03 ^A^	2.19 ^A^	1.83 ^A^	0.30	0.630	<0.001	0.852
7 d postpartum	1.87 ^A^	1.41 ^A^	1.86 ^A^	1.73 ^A^	0.28			
21 d postpartum	1.26 ^B^	1.10 ^B^	1.20 ^B^	1.34 ^B^	0.17			
TAS [mmol/L]								
Baseline (0 d)	1.11	1.12	1.09	1.14	0.05			
End suppl. (21 d)	1.10	1.14	1.14	1.09	0.04	0.691	0.192	0.567
7 d postpartum	1.18	1.12	1.18	1.14	0.05			
21 d postpartum	1.26	1.16	1.12	1.10	0.08			

^1^ Mix oil consists of flaxseed oil and BSFL oil in a 2:1 ratio. Baseline values are arithmetic means (n = 8 per treatment), whereas post-baseline values are baseline-adjusted least-squares means. The largest SEM among the four treatment means is presented for each sampling time. *p*-values refer to treatment, time, and treatment × time effects during the post-baseline period, with the corresponding baseline value included as a covariate. AST, GGT, ALP, and TBIL were analyzed after natural-log transformation; back-transformed least-squares means are presented. For GGT, treatment × baseline terms were included because the homogeneity-of-regression-slopes assumption was not met. When the treatment × time interaction was not significant, uppercase superscripts indicate differences among marginal time means. When the interaction was significant, lowercase superscripts indicate treatment differences within a sampling time, and uppercase superscripts indicate time differences within a treatment (Holm-adjusted *p* < 0.05). AST, aspartate aminotransferase; ALT, alanine aminotransferase; GGT, gamma-glutamyl transferase; ALP, alkaline phosphatase; TBIL, total bilirubin; TAS, total antioxidant status.

**Table 8:** Serum protein and renal-function indicators in ewes across the experimental period.

Blood Parameters	Treatments				SEM	*p*-Value		
	Control	Flaxseed Oil	BSFL Oil	Mix ^1^ Oil		Treatment	Time	Treatment × Time
TP [g/L]								
Baseline (0 d)	56.83	62.03	62.19	64.12	4.02			
End suppl. (21 d)	52.84	59.12	58.74	58.27	2.53	0.069	0.251	0.814
7 d postpartum	51.44	57.34	54.50	55.53	2.49			
21 d postpartum	49.83	60.50	53.09	57.18	3.19			
ALB [g/L]								
Baseline (0 d)	27.27	26.55	25.57	26.51	1.26			
End suppl. (21 d)	26.66 ^A^	27.69 ^A^	29.17 ^A^	27.01 ^A^	0.85	0.808	<0.001	0.327
7 d postpartum	27.71 ^A^	26.85 ^A^	27.46 ^A^	27.05 ^A^	0.93			
21 d postpartum	23.42 ^B^	24.95 ^B^	23.94 ^B^	24.76 ^B^	1.00			
BUN [mmol/L]								
Baseline (0 d)	4.01	4.42	5.05	5.11	0.41			
End suppl. (21 d)	4.43 ^A^	4.29 ^A^	4.68 ^A^	3.79 ^A^	0.38	0.838	0.014	0.617
7 d postpartum	3.19 ^B^	3.88 ^B^	3.38 ^B^	3.38 ^B^	0.39			
21 d postpartum	4.36 ^AB^	3.78 ^AB^	3.91 ^AB^	3.91 ^AB^	0.57			
CREA [µmol/L]								
Baseline (0 d)	91.83	82.99	84.34	97.92	4.28			
End suppl. (21 d)	92.24 ^bA^	90.44 ^b^	106.08 ^aA^	111.39 ^aA^	3.62	0.012	<0.001	0.030
7 d postpartum	74.66 ^B^	91.61	83.08 ^B^	86.50 ^B^	5.44			
21 d postpartum	73.59 ^B^	88.15	86.97 ^B^	82.96 ^B^	4.19			

^1^ Mix oil consists of flaxseed oil and BSFL oil in a 2:1 ratio. Baseline values are arithmetic means (n = 8 per treatment), whereas post-baseline values are baseline-adjusted least-squares means. The largest SEM among the four treatment means is presented for each sampling time. *p*-values refer to treatment, time, and treatment × time effects during the post-baseline period, with the corresponding baseline value included as a covariate. BUN was analyzed after natural-log transformation; back-transformed least-squares means are presented. When the treatment × time interaction was not significant, uppercase superscripts indicate differences among marginal time means. When the interaction was significant, lowercase superscripts indicate treatment differences within a sampling time, and uppercase superscripts indicate time differences within a treatment (Holm-adjusted *p* < 0.05). TP, total protein; ALB, albumin; BUN, blood urea nitrogen; CREA, creatinine.

## Data Availability

The datasets used and analyzed during the current study are available from the corresponding author on reasonable request.

## Abbreviations

The following abbreviations are used in this manuscript:

GC–MS	gas chromatography–mass spectrometry
AST	aspartate aminotransferase
ALT	alanine aminotransferase
ALP	alkaline phosphatase
BHB	β-hydroxybutyrate
BUN	blood urea nitrogen
CREA	creatinine
GGT	γ-glutamyl transferase
GLU	glucose
HDL-C	high-density lipoprotein cholesterol
LDL-C	low-density lipoprotein cholesterol
NEFA	non-esterified fatty acids
SNF	solids-not-fat
TAS	total antioxidant status
TBIL	total bilirubin
TC	total cholesterol
TP	total protein
TG	triglycerides
BSFL oil	black soldier fly larvae oil
BSF	black soldier fly
FA	fatty acid
ALA	α-linolenic acid
ARA	arachidonic acid
CLA	conjugated linoleic acid
VFA	volatile fatty acids
SFA	saturated fatty acids
UFA	unsaturated fatty acids
MUFA	monounsaturated fatty acids
PUFA	polyunsaturated fatty acids
OCFA	odd-chain fatty acids
BCFA	branched-chain fatty acids
OBCFA	odd- and branched-chain fatty acids
MCFA	medium-chain fatty acids
LCFA	long-chain fatty acids
AI	atherogenicity index
TI	thrombogenicity index
